# Genome Sequence of *Delftia acidovorans* HK171, a Nematicidal Bacterium Isolated from Tomato Roots

**DOI:** 10.1128/genomeA.01746-16

**Published:** 2017-03-02

**Authors:** Jae Woo Han, Mira Oh, Gyung Ja Choi, Hun Kim

**Affiliations:** a Center for Eco-Friendly New Materials, Korea Research Institute of Chemical Technology, Daejeon, South Korea; b Department of Green Chemistry and Environmental Biotechnology, Korea University of Science and Technology, Daejeon, South Korea

## Abstract

*Delftia acidovorans* strain HK171, isolated from tomato roots, exhibited nematicidal activity against *Meloidogyne incognita*. Here, we present the genome sequence of *D. acidovorans* strain HK171, which consists of one circular chromosome of 6,430,384 bp, with 66.9% G+C content.

## GENOME ANNOUNCEMENT

The genus *Delftia* phylogenetically belongs to the *Comamonadaceae* family of the betaproteobacteria, which are found in seawater, soil, plants, and activated sludge (1). They were often reported to be able to degrade or transform various kinds of organic and inorganic compounds (2). Some strains of *Delftia* have been reported as plant growth-promoting bacteria or biocontrol agents (3, 4). In our study, *Delftia acidovorans* HK171 strain was originally isolated from the roots of a tomato plant, exhibiting nematicidal activity against the root-knot nematode *Meloidogyne incognita*.

Genomic DNA of the HK171 strain was extracted by QIAamp genomic DNA kits (Qiagen, Hilden, Germany). The purity and concentration of the genomic DNA were determined using an Agilent 2100 Bioanalyzer (Palo Alto, CA, USA). The PacBio RSII single-molecule real-time (SMRT) sequencing (Pacific Biosciences, Menlo Park, CA, USA) generated a total of 1,432,240,037 nucleotides according to 190,066 reads (*N*_50_, 10,426 bp; mean subread length, 7,535 bp), with 223-fold coverage of the genome. The subreads were assembled *de novo* using the RS Hierarchical Genome Assembly Process 3.0 in the SMRT Analysis program 2.3 (Pacific Biosciences) (5). Genome annotation was predicted using Prokka 1.11 and Prokaryotic Genome Annotation Pipeline 3.3 of NCBI (6, 7). Average nucleotide identities (ANI) with the currently available genome sequences of *Delftia* spp. were calculated using Oat 0.93 (8).

The whole genome of the HK171 strain features a single circular chromosome which is 6,430,384 bp in length with 66.9% G+C content, and with no plasmids detected. In total, 5,468 coding DNA regions were identified, with 81 tRNA, 15 rRNA, three noncoding RNA (ncRNA), and 86 pseudogenes. All five 16S rRNA genes of the HK171 strain were 100% identical to each other. The 16S rRNA gene sequence alignment on the EzTaxon server (http://www.ezbiocloud.net/identify) revealed that the HK171 strain belonged to the genus *Delftia* and was most closely related to *D. tsuruhatensis* NBRC 16741^T^ (accession no. BCTO01000107.1) and *D. acidovorans* 2167^T^ (accession no. JOUB01000005.1), with 99.86 and 99.66% similarity, respectively. Additionally, the HK171 strain shared very high (>97.5%) average nucleotide identity (ANI) values with *D. acidovorans* Cs1-4 (accession no. CP002735.1), *D. acidovorans* 2167 (accession no. KN046795.1), and *D. acidovorans* SPH-1 (accession no. CP000884.1) at the genome level.

The genome of the HK171 strain contains seven serine protease, three metalloprotease, and 24 protease-related genes. Several reports have described that the secretion of extracellular proteinases plays an important role in the nematicidal activity of antagonistic strains of *Bacillus*, *Pseudomonas*, and *Stenotrophomonas* (9–11). We also found that the genome contains gene loci for resorcinol, terpene, arylpolyene, siderophore, and thiazole/oxazole-modified microcin (TOMM) identified by antiSMASH analysis (12); various resorcinol compounds and plantazolicin (a member of the TOMM family) have been known to be involved in nematicidal activity (13–15). Overall, the whole-genome analysis of *D. acidovorans* strain HK171 provides a genetic foundation for the understanding of biocontrol mechanism to root-knot nematode.

### Accession number(s).

The genome sequence of *D. acidovorans* HK171 strain has been deposited into the GenBank database under the accession number CP018101.
